# Diabetes and weight gain after bariatric surgery, due to Cushing's syndrome

**DOI:** 10.1186/1758-5996-7-S1-A97

**Published:** 2015-11-11

**Authors:** Vagner Rosa Bizarro, Lis Marina Mesquita Araujo, Julio Cesar Salles Santos, Alessandre Gomes Lima, Paola Calafiori Resende, Ana Luísa Conceição de Jesus, Theara Castro Nicolau, Denise Rosso Tenório Wanderley Rocha, André Rocha Jorge, Alberto Krayyem Arbex

**Affiliations:** 1 IPEMED-Instituto de Pesquisa e Ensino Médico, Rio de Janeiro, Brazil

## Background

The term Cushing's syndrome (SC) describes a condition resulting from prolonged exposure to excessive glucocorticoids. The routine use of abdominal image procedures has significantly increased the incidental finding of adrenal masses. A documentation of the presence of endogenous hypercortisolism is made with salivary, urinary or serum cortisol measurements, using samples collected with appropriate timing and/or after the use of low doses (1mg) of dexamethasone.

## Materials and methods

E.A.R, 45 yrs. old, female, referred to our Service in 2013, due to the presence of a tumor in right adrenal discovered in abdominal TC. This exam was realized as a routine AFTER bariatric surgery (BS) in 2004. She told us that she lost 40 Kg after surgery. However, in 6 months her weight gained 20Kg with no clear reason. Increased blood pressure and hyperglycemia appeared. She related pain in limbs, alopecy and amenorrheia, a year ago. In use of: Losartan 100mg/day, Spironolactone 200mg/day, Carvedilol 25mg/day, Aspirin 100mg/day, Simvastatin 20mg/day, Omeprazol 20mg/day, Furosemide 40mg/day. Physical exam: Weight: 89Kg, heigh: 1,58m, BMI: 35,6Kg/m^2^, WC: 116cm, Blood pressure: 200x120mmHg, HR: 104bpm. Proximal muscle weakness, abdominal striae, moon face, hump back, blurred vision, neurological, musculoskeletal, skin and hearing alterations. Results: A1c: 8%( N<5.7), TSH: 0,7mUI/L (N: 0,3-4,2), ACTH: 18,8pg/mL (N: 7,2-63,3), basal cortisol: 24,5mcgmL (N: 7-28), Aldosterone: 7ng/dl(4-31), androstenidione: 1,5ng/dl (N: 0,8-4,4), catecolamins: 319mcg/24hs (N: 190-450), urinary cortisol: 1089mcg/24hs(N: 10-90), DHEA: <15mmol/L (N: 0,7-6,75), estradiol: 19,6ng/dl (N: <3), renine: 4,9ng/mL/h (up, N: 1,5-5,7), testosterone total: <12pgmL (N: 0,3-2,5), FSH: 11mUImL (menopause N: >30), LH: 12,1mUImL (N: >15), prolactin: 10,3mcg/L (N: 2-15), supression test after 1mg of dexamethasone->cortisol: 22,48mcg/dl (N<1,8). We started Metformin 1700mg/day, Cetoconazol 800mg/dia and Insulin NPH 40UI/day. Referred to surgery.

## Conclusion

Since undiagnosed CS might result in severe perioperative complications in patients already at increased risk, this case report underlines the importance of a careful endocrine evaluation of morbidly obese patients. Obese subjects scheduled for BS may reveal undiagnosed dysfunctions that require specific therapy and/or contraindicate the surgical treatment. Such Results may help to define the extent of the endocrinological screening to be performed in obese patients undergoing BS.

